# Circulation first – the time has come to question the sequencing of care in the ABCs of trauma; an American Association for the Surgery of Trauma multicenter trial

**DOI:** 10.1186/s13017-018-0168-3

**Published:** 2018-02-05

**Authors:** Paula Ferrada, Rachael A. Callcut, David J. Skarupa, Therese M. Duane, Alberto Garcia, Kenji Inaba, Desmond Khor, Vincent Anto, Jason Sperry, David Turay, Rachel M. Nygaard, Martin A. Schreiber, Toby Enniss, Michelle McNutt, Herb Phelan, Kira Smith, Forrest O. Moore, Irene Tabas, Joseph Dubose, AAST Multi-Institutional Trials Committee

**Affiliations:** 1 Trauma, Emergency surgery and Critical Care, Virginia Commonwealth University, 417 N 11th St, Richmond, VA 23298, Richmond, VA 23298-0454 USA; 2 University of California San Francisco, San Francisco, USA; 3 University of Florida College of Medicine, Gainesville, USA; 4 John Peter Smith Hospital Network, Fort Worth, USA; 5 Centro de Investigaciones Clínicas, Fundación Valle del Lili Hospital, Cali, Colombia; 6 University of Southern California, California, USA; 7 University of Pittsburg, Pittsburg, USA; 8 Loma Linda University, Loma Linda, USA; 9 Hennepin County Medical Center, Minneapolis, USA; 10 Oregon Health & Science University, Portland, USA; 11 University of Utah School Medicine, Salt Lake City, USA; 12 McGovern Medical School at the University of Texas Health Science Center at Houston, Houston, USA; 13 University of Texas-Southwestern Medical Center, Dallas, USA; 14 Chandler Regional Medical Center, Chandler, USA; 15 Dell Medical School, University of Texas at Austin, Austin, USA; 16 Shock Trauma Centre, University of Maryland, Baltimore, USA

**Keywords:** Trauma resuscitation, Circulation first, Effects of intubation, Resuscitation in trauma, Trauma, Resuscitation, Circulation, Hypovolemia and hypotension, Hypotension in trauma, Hypotension and resuscitation

## Abstract

**Background:**

The traditional sequence of trauma care: Airway, Breathing, Circulation (ABC) has been practiced for many years. It became the standard of care despite the lack of scientific evidence. We hypothesized that patients in hypovolemic shock would have comparable outcomes with initiation of bleeding treatment (transfusion) prior to intubation (CAB), compared to those patients treated with the traditional ABC sequence.

**Methods:**

This study was sponsored by the American Association for the Surgery of Trauma multicenter trials committee. We performed a retrospective analysis of all patients that presented to trauma centers with presumptive hypovolemic shock indicated by pre-hospital or emergency department hypotension and need for intubation from January 1, 2014 to July 1, 2016. Data collected included demographics, timing of intubation, vital signs before and after intubation, timing of the blood transfusion initiation related to intubation, and outcomes.

**Results:**

From 440 patients that met inclusion criteria, 245 (55.7%) received intravenous blood product resuscitation first (CAB), and 195 (44.3%) were intubated before any resuscitation was started (ABC). There was no difference in ISS, mechanism, or comorbidities. Those intubated prior to receiving transfusion had a lower GCS than those with transfusion initiation prior to intubation (ABC: 4, CAB:9, *p* = 0.005). Although mortality was high in both groups, there was no statistically significant difference (CAB 47% and ABC 50%). In multivariate analysis, initial SBP and initial GCS were the only independent predictors of death.

**Conclusion:**

The current study highlights that many trauma centers are already initiating circulation first prior to intubation when treating hypovolemic shock (CAB), even in patients with a low GCS. This practice was not associated with an increased mortality. Further prospective investigation is warranted.

**Trial registration:**

IRB approval number: HM20006627. Retrospective trial not registered.

## Background

The evidence supporting the systematic Airway, Breathing, and Circulation (ABC) approach to injured patients is based on expert consensus with little literature to support the clinical application of the order in which this sequence should be applied [1]. Early intubation can result in deleterious effects in adult and pediatric patients with traumatic brain injury [2–6]. There are many physiological explanations why intubation in hypovolemic shock might result in worse perfusion [2–6]. After rapid sequence intubation (RSI), there is a vasodilatory response placing the hypovolemic patient at very high risk for more pronounced hypotension and decreased perfusion [7–11]. Shafi et al. previously published an analysis of the national trauma data bank showing how pre-hospital intubations resulted in further hypotension in hypovolemic patients [12]. In addition to the vasodilation following RSI, positive pressure ventilation decreases venous return and therefore cardiac output resulting in further decreased perfusion [13]. This event is critical in hypovolemic patients that are dependent on venous return and adrenergic response to maintain perfusion [14–17].

In the medical literature, while treating patients with cardiorespiratory arrest, the focus of the protocols have moved from acquiring an airway first, to prioritizing perfusion by initiating chest compressions expeditiously [14, 15, 18, 19]. This has resulted in better outcomes [14, 15, 18, 19]. This practice has not been previously investigated in the trauma population.

The objective of this study is to investigate if there are differences in outcome when following the traditional sequence of ABC versus starting transfusion and resuscitation first (CAB). We hypothesized that patients in hypovolemic shock would have at least comparable outcomes with initiation of bleeding treatment (transfusion) prior to intubation, compared to those patients treated with the traditional ABC sequence.

## Methods

The present study was sponsored by the American Association for the Surgery of Trauma (AAST) multicenter trials committee, and 12 level one trauma centers contributed patients to the study. The study was approved by the Institutional Review Board of each participating site. We performed a retrospective analysis of all patients that presented to trauma centers with presumptive hypovolemic shock (report of pre-hospital hypotension or confirmed hypotension on arrival to the emergency department) and required intubation in the trauma bay from January 1, 2014 to July 1, 2016.

Data collected included demographics, comorbidities (hypertension, chronic obstructive pulmonary disease, history of stroke, congestive heart failure, diabetes, chronic renal failure, others), pre-hospital intravenous fluids, timing of intubation, vital signs before and after intubation, and the order of initiation of blood products to intubation. Patients were classified in the ABC group if they were intubated before packed red blood cell transfusion was started. Patients were considered in the CBA group if transfusion was begun before intubation medications were given. Massive transfusion was defined as receiving 10 units of packed red blood cells in the first 24 h. Univariate and multivariate predictors of mortality were determined using mixed effects logistic regression controlling for center effect. Univariate predictors at the *p* < 0.05 level of significance and clinically significant variables were considered in the multivariate models. Subset analysis was performed of all patients with a confirmed systolic blood pressure of 90 mmHg or less in the emergency department prior to intubation, penetrating mechanism, initial GCS < =8, and need for massive transfusion. Continuous variables are reported as medians (interquartile range). All analyses were conducted with STATA v14.2 (College Station, TX).

## Results

Twelve centers were included in the study, including an international trauma program. During the study period, 440 patients met inclusion criteria of either a reported hypotensive episode in the field or confirmed hypotension in the emergency department and need for intubation. The cohort median age was 39 (26–54) with 33.6% suffering from penetrating mechanisms. The group was severely injured with a median initial emergency department SBP (systolic blood pressure) of 80 mmHg (59–98 mmHg), initial GCS 6 [3–14], and a median injury severity score (ISS) of 25 (16–38). Comorbidities were common with 42.1% of the cohort having one or more known comorbidities. Median hospital length of stay was 6 days [1–20]. Overall mortality was 49.1%.

The CAB group consisted of 245 (55.7%) who received intravenous blood product resuscitation first, and the ABC group, 195 (44.3%), representing those intubated before any resuscitation was started. There was no difference in age, ISS, mechanism, or comorbidities between the groups (Table 1). Patients in the CAB group had an average GCS of 9 compared with 4 in the ABC group, *p* = 0.0005 (Table 1).

**Table 1:** Demographics of the CAB vs. ABC group

	*n*	CAB	ABC	*p* value
Median age (years, IQR)	440	41 (28–56)	37 (25–53)	0.26
Median ISS (IQR)	440	25 (16–38)	25 (17–38)	0.99
Penetrating mechanism	440	30.8 %	35.5 %	0.3
Hypertension	440	11.8 %	10.6 %	0.7
COPD	440	1.5 %	2.0 %	0.7
CAD	440	1.0 %	3.3 %	0.1
CVA	440	0.0 %	2.0 %	0.05
CHF	440	0.5 %	1.2 %	0.44
DM	440	5.6 %	3.7 %	0.33
CRF	440	0.0 %	1.2 %	0.12
Other comorbidity	440	33.3 %	33.5 %	0.98
No comorbidities	440	56.9 %	58.0 %	0.83
Initial GCS	434	9 (3–15)	4 (3–13)	0.0005

Abbreviations: *CAB* Transfusion prior to intubation, *ABC* Intubation prior to transfusion, *ISS* injury severity score, *IQR* Interquartile range, *COPD* Chronic obstructive pulmonary disease, *CAD* Coronary artery disease, *CVA* Stroke, *CHF* Congestive heart failure, *DM* Diabetes mellitus, *CRF* Chronic renal failure, *GCS* Glasgow coma score

The percentage of patients receiving pre-hospital IVFs (intravenous fluids) and the amount received were similar (CAB 500 mL vs. ABC 800 mL, *p* = 0.13; Table 2). The only difference in hemodynamic parameters prior to intubation was a lower initial emergency department diastolic blood pressure in the CAB group (48 vs 51 mmHg, *p* = 0.03). Pre-intubation SBP (systolic blood pressure) and DBP (diastolic blood pressure) were the same (Table 2). Although only half of the cohort had a pre-intubation lactate obtained, there was no difference between the groups (Table 2). Following intubation, there was no difference in blood pressure or lactate between the two groups (Table 2).

**Table 2:** Pre-hospital and emergency department fluids, vital signs, and labs

	*n*	CAB	ABC	*p* value
IVF pre-hospital	440	53.9 %	55.1 %	0.79
IVF volume pre-hospital (mL)	151	500 (250–1010)	800 (300–1800)	0.13
SBP initial	440	80 (50–95)	82 (62–99)	0.1
DBP initial	422	48 (0–64)	51 (25–68)	0.03
SBP pre-intubation	440	84 (54–101)	85 (62–99)	0.3
DBP pre-intubation	430	50 (0–66)	53 (0–76)	0.07
SBP post-intubation	439	92 (42–114)	90 (62.5–113.5)	0.53
DBP post-intubation	434	52.5 (20–73)	58 (32–79)	0.11
Lactate pre-intubation	222	0 (0–3)	0 (0–2)	0.5
Lactate post-intubation	325	3 (0–6)	2 (0–5)	0.12

All values represent medians (interquartile range)

Abbreviations: *IVF* Intravenous fluids, *mL* Milliliter, *SBP* Systolic blood pressure, *DBP* Diastolic blood pressure

Both groups had a similar percentage of patients that received blood transfusion overall (CAB 62.1% vs. ABC 69.4% *p* = 0.11) and there was no difference in those receiving massive transfusion (Table 3). There was no statistical difference regarding those surviving to ICU admission with 72.8 and 67.8% admitted initially to the ICU in each group (Table 3). The median LOS in the CAB was slightly longer at 8 days compared with 4 days, but the difference did not reach statistical significance (*p* = 0.24). There was also no statistically significant difference in mortality between groups (CAB 47% and ABC 50%, *p* = 0.63).

**Table 3:** Outcomes of the CAB and ABC groups

	*n*	CAB	ABC	*p* value
Transfusion in first 24 h	440	62.1 %	69.4 %	0.11
Massive transfusion	440	34.4 %	29.4 %	0.27
ICU admission	440	72.8 %	67.8 %	0.25
LOS*	440	8 (0–22)	4 (1–20)	0.24
Mortality	440	47.7 %	50.0%	0.63

*Median (interquartile range)

*ICU* Intensive care unit, *LOS* Length of stay

In mixed effects logistic regression controlling for center effect (*n* = 416 patients), initial GCS (OR 0.76, 0.72–0.80, *p* < 0.0001) and emergency department SBP were the only independent predictors of death (0.97, 0.96–0.98, *p* < 0.0001). Intubation before initiation of transfusion (*p* = 0.13) and emergency department DBP (*p* = 0.17) were not independent predictors of mortality. In the subset analyses of patients with confirmed initial hypotension (SBP ≤ 90 mmHg) in the emergency department, penetrating mechanism, initial GCS ≤ 8, or requiring massive transfusion, intubation before transfusion (ABC) was not an independent predictor of mortality (Table 4).

**Table 4:** Independent predictors of mortality in subset analysis

	Subset of interest							
	Initial SBP ≤ 90		Penetrating		Initial GCS ≤ 8		Massive transfusion	
	(*n* = 302)		(*n* = 140)		(*n* = 246)		(*n* = 126)	
Variable	OR (95% CI)	*p* value	OR (95% CI)	*p* value	OR (95% CI)	*p* value	OR (95% CI)	*p* value
Initial SBP	0.97 (0.96–0.99)	< 0.001	0.99 (0.96–1.01)	0.456	0.98 (0.97–0.99)	0.008	0.97 (0.95–0.99)	0.008
Initial DBP	1.00 (0.99–1.02)	0.644	0.98 (0.95–1.02)	0.377	1.00 (0.99–1.02)	0.603	1.02 (1.00–1.04)	0.061
Initial GCS	0.77 (0.72–0.82)	< 0.001	0.65 (0.56–0.75)	< 0.001	0.57 (0.46–0.70)	< 0.001	0.79 (0.72–0.87)	< 0.001
ABC	0.57 (0.29–1.13)	0.108	0.79 (0.22–2.9)	0.721	0.57 (0.28–1.16)	0.120	0.52 (0.20–1.36)	0.183

## Discussion

For patients in extremis including those suffering cardiac arrest, airway, then breathing, followed by circulation have been the priorities established in resuscitative algorithms including the Advanced Trauma Life Support ATLS course. However, more recent data in non-trauma patients have found that prioritizing perfusion over airway has been associated with better outcomes in patients with a primary cardiac event [15, 18–20]. There are a number of potential explanations including the phenomenon of agonal breaths or gasping that happens in patients in extremis which has been shown to increase cardiac output and perfusion [21–23].

The idea of prioritizing circulation over airway in the trauma cohort has not been previously investigated. In part, this is difficult to study in a prospective fashion as it is well established that even brief periods of hypoxemia portend a poor prognosis in brain-injured patients or those with a secondary brain injury from cardiac arrest or profound hypotension [4, 20]. Most importantly, it is difficult in the first moments of emergency department evaluation to determine if a patient in extremis, especially a bluntly injured patient, has both significant bleeding and traumatic brain injury. However, the risk of intubation in hypovolemic patients is worsening hypotension which also has deleterious effects especially in the brain-injured patient.

During intubation, administration of sedative and neuromuscular relaxants result in vasodilation counteracting the very much needed adrenergic response keeping the patient in profound hypovolemic shock alive [7–11]. Even if a patient does not experience a hemodynamic collapse after this vasodilation, then the positive pressure ventilation can further decrease the venous return and cardiac output resulting in cardiac arrest [13, 17]. It is plausible that there may be a benefit to redefining the classic ABC sequence taught in ATLS for the patient felt to be presenting in shock as intubation and positive pressure ventilation can result in further physiological insult [24]. Prioritizing resuscitation, or at least not causing further physiological challenges, can be desirable to ensure perfusion [12, 24].

In this retrospective study, it was surprising with the emphasis on ABC in ATLS to find that the use of transfusion prior to intubation occurred in the majority of patients (55.7%). In a retrospective study, it is impossible to understand clinician decision making regarding why these patients were resuscitated with a CAB sequence rather than ABC. However, it is likely this is a reflection of the evolution of massive transfusion protocols and practices that have become wide spread in the last 5–10 years. It is now well established that time to initiation of massive transfusion protocols in those patients suffering from hemorrhagic shock is a major determinant of outcome. As a result, it is now common place in level one trauma centers to have rapid and immediate access to blood products including some centers storing these products directly in the trauma bay or emergency department. This allows for extremely early initiation of transfusion without delaying intubation.

Our results have demonstrated that initiation of transfusion prior to intubation is associated with equivalent mortality outcome compared with the concept of airway first (ABC). It is plausible that those patients in the CAB group had more obvious signs of hypovolemic shock that were not possible to ascertain using retrospective data. Thus, transfusion was begun in rapid fashion which preceded intubation. In an effort to further elucidate if particular types of patients were more or less likely to benefit from CAB, multiple subset analyses were undertaken. There was no difference in outcome demonstrated for those with hypotension on arrival to the emergency department, those suffering an initial decreased GCS, penetrating trauma, or those receiving massive transfusion. The concept of simultaneous ABCs might not be possible in places where one provider is responsible for the entire care of a bleeding trauma patient [25, 26]. It also might not be clear for clinicians working in areas in which trauma is not a prevalent disease. Especially, since the international guidelines for trauma care traditionally teach that the sequence of ABC is life saving and should be followed systematically on the strict order airway, breathing, circulation [27].

### Limitations

The present series has several limitations including the use of retrospective data; this fact can offer obvious bias. Extraction of data from medical records review did not allow identification of reasoning for transfusion before intubation. We aimed to include patients with hypotension due to hypovolemia, not patients with hypotension for other reasons such as pneumothorax, blunt cardiac injury, or pericardial tamponade.

Furthermore, pre-hospital vital sign records were not universally available, and therefore, patients were included in the study if they were called hypotensive in the field or had hypotension in the emergency department. Given that a number of patients were not hypotensive on arrival to the emergency department, a subset analysis of those with an initial SBP ≤ 90 mmHg was performed. These results remained unchanged compared with the entire cohort results. It was also not possible to determine the neurologic outcome of patients surviving to discharge, and therefore, we cannot determine if CAB had a negative impact on functional outcome.

The mortality rate of 47.7 and 50% for an ISS of 25 (median) is high for both groups due to the degree of injury and the rate of penetrating trauma (> 30% in both groups). Given the lack of inferiority of CAB compared with the ABC group for mortality outcome, early initiation of transfusion while not delaying intubation may have promise for improving trauma outcomes further. However, to ideally understand the true impact of intubation on hypovolemic patients a prospective observational trial needs to be developed to fully elucidate if CAB offers an advantage similar to that seen in medical patients experiencing cardiac arrest.

## Conclusions

In this retrospective review, national and international centers are already addressing circulation first before airway in bleeding trauma patients. A prospective, multicenter trial should be the next step to understand the physiological challenges of intubation in hypovolemic patients.
